# Adverse childhood experiences and mental health issues in patients seeking cosmetic surgery: A case-control study

**DOI:** 10.1016/j.jpra.2025.03.010

**Published:** 2025-03-25

**Authors:** Fateme Faezi, Sohrab Amiri

**Affiliations:** a Department of General Psychology, Faculty of Literature, Humanities, and Social Sciences, Islamic Azad University،Science And Research Branch, Tehran, Iran.; b Spiritual Health Research Centre, Life Style Institute, Baqiyatallah University of Medical Sciences, Tehran, Iran

**Keywords:** Adverse childhood experiences, Cosmetic surgery, Mental health issues, Self-esteem

## Abstract

**Objectives:**

Cosmetic surgery has emerged as a notable phenomenon within the health industry, with significant advancements, particularly in the recent decades. This study sought to explore the prevalence of mental health issues and adverse childhood experiences among individuals pursuing cosmetic surgery.

**Methods:**

This case-control study included women seeking cosmetic surgery as the case group, while women from the general population were included in the control group. Each group consisted of 100 participants. The research was conducted between May and June 2024, during which relevant data were collected. The study assessed key outcome variables, including adverse childhood experiences, self-rated health, self-esteem, and general health indicators such as depression, anxiety, social dysfunction, and somatic complaints. The analysis employed adjusted multivariate covariance with 95% confidence intervals.

**Results:**

The multivariate covariance analysis revealed significant differences between the cosmetic surgery and control groups. Pairwise comparisons indicated that individuals in the cosmetic surgery group reported higher rates of emotional abuse (P < 0.001), physical abuse (P < 0.001), sexual abuse (P < 0.001), emotional neglect (P < 0.001), and physical neglect (P < 0.001). Moreover, this group exhibited lower levels of self-esteem (P < 0.001) and higher occurrences of somatic complaints (P < 0.001), anxiety (P < 0.001), and depression (P < 0.001) compared to the control group.

**Discussion:**

This study highlights that individuals seeking cosmetic surgery are more likely to have experienced adverse childhood events and faced mental health issues than those in the general population. These findings suggest that adverse childhood experiences may contribute to the decision to undergo cosmetic surgery later in life. Consequently, addressing mental health issues in greater depth and precision is essential in this context.

## Introduction

Cosmetic surgery has emerged as a significant phenomenon in the healthcare field, and it has been gaining momentum in the recent decades due to the advancements in medical technology and it is defined as “surgery to alter the appearance of the body, in the absence of disease, injury, or congenital deformity”.^1^ The global prevalence of cosmetic procedures has been steadily rising each year.^2^ In 2022, liposuction was the most commonly performed surgical cosmetic procedure worldwide, followed closely by breast augmentation. Among the nonsurgical procedures, botulinum toxin (commonly known as Botox) ranked first, with hyaluronic acid-based (dermal) fillers coming second.^3^ The United States recorded the highest number of cosmetic procedures globally in 2022, accounting for 7.4 million procedures, followed by Brazil, where more than 3 million procedures occurred annually.^3^ Despite the associated risks and health implications, cosmetic surgery has increasingly become a societal norm.^4^ Understanding factors contributing to unsuccessful outcomes is pivotal for ensuring better surgical results.^5^ These factors include physical and psychological elements.^6^

The link between cosmetic surgery and psychiatric disorders is not yet fully established.^6^ Estimates from certain studies suggest that 30%-70% of the individuals seeking cosmetic surgery may have some form of psychiatric disorder.^7^ Consequently, psychological evaluations are recommended before performing surgery to minimize unfavorable outcomes for patients and surgeons.^8^ Research highlights a significantly higher prevalence of body dysmorphic disorder (BDD) among cosmetic surgery seekers—20% compared to 0.7%-2.4% in the general population.^5^^,^^9^ Patients with BDD are often at risk of undergoing excessive surgeries and frequently express dissatisfaction postprocedure.^10^ Moreover, those pursuing cosmetic surgery commonly report mental health issues such as depression, anxiety, and eating disorders.^11^ Pre-existing mental health conditions can adversely influence the postsurgery outcomes, with studies showing that cosmetic surgery does not necessarily lead to positive psychological results.^12^^,^^13^ Additionally, some individuals or demographic groups may have a predisposition to unsatisfactory outcomes following surgery.^14^ It has also been observed that patients with mental health problems have a greater need for acute care after cosmetic procedures.^15^ Given these findings, assessing the mental health status of individuals seeking cosmetic surgery is crucial; although some studies have explored this area in depth, much remains to be uncovered.^6^ Particularly, studies have increasingly emphasized that early life events significantly shape health outcomes throughout one's life.^16^

Adverse childhood experiences (ACEs) provide valuable insight into the underlying motivations for pursuing cosmetic surgery; however, this aspect has received minimal investigation to date. BDD—a prevalent concern among cosmetic surgery seekers^5^—has been strongly linked to these adverse experiences during childhood.^17^ ACEs encompass a range of traumatic events such as abuse, neglect, and dysfunctional family dynamics^18^, all of which can profoundly impact multiple dimensions of health in later life.^16^ This study aimed to explore the intersection of mental health issues and ACEs among individuals seeking cosmetic surgery to better understand the psychological underpinnings influencing their decisions.

## Methods

### Study design and population

The study used a case-control and cross-sectional design, where women seeking cosmetic surgery were designated as the case group, and women from the general population served as the control group. For the case group, a prominent specialized clinic in Tehran, Iran, that caters to a high volume of clients was selected. Prior to commencing the study, coordination with this clinic was established, and a written agreement was secured for the collaboration. Participants in the case group were recruited through convenience sampling at the clinic. In contrast, participants for the control group were recruited online using Google Forms. Various approaches were employed to invite the control group to join the study. Some participants were friends or acquaintances of cosmetic surgery seekers who neither expressed interest in cosmetic procedures nor had undergone any in the past. Others included university students who received the participation link via student group communications and were eligible to partake if they met the criteria. Most participants in the control group were identified through acquaintances of cosmetic surgery seekers, as their contact information was readily accessible. Data collection for the case group was conducted in person, whereas for the control group, it occurred entirely online. The pool of potential participants exceeded the required sample size, allowing for random sampling to finalize participant selection. Ultimately, the study included 200 participants, with 100 individuals in the case and control groups. The research was conducted between May and June 2024, during which data were collected systematically.

### Ethical considerations

This study adhered to ethical standards in accordance with the Helsinki Declaration^19^ and received approval from the local ethics committee (IR.IAU.SRB.REC.1403.232).

### Consent to participate

To participate in the study, each participant signed an informed consent form.

### Sample size

The sample size calculation was conducted using G*Power software version^20^ was used for sample size calculation. With Tail one, an effect size of 0.50, a power of 0.95, α error probability of 0.05, and allocation ratio of 1, the required sample size was determined to be 88 participants per group.^20^ To account for potential missing data and minimize the associated errors, the minimum sample size for the case and control groups was increased to 100 participants.

### Eligibility criteria

The inclusion criteria were as follows: 1) Women who pursued cosmetic surgery which is defined below, and 2) women aged 18 years or older.

The exclusion criteria included women who self-reported substance abuse or psychiatric disorders.

### Definition

To identify the cases examined in this research, specific criteria were considered. These criteria highlight the pursuit of extreme cosmetic surgery and include individuals who, even when such procedures were unnecessary or unfeasible, persisted in seeking them, as defined in other contexts.^21^

## Instruments

### Sociodemographic

A checklist was employed by the authors to gather demographic and health information through participant self-reports. This included details such as age, education, economic status, medical history, body mass index, smoking habits, and alcohol consumption.

### General Health Questionnaire

The General Health Questionnaire serves as a screening instrument designed to identify minor psychiatric disorders.^22^ Comprising 28 items, it evaluates 4 distinct symptom categories: somatic symptoms (items 1–7), anxiety and insomnia (items 8–14), social dysfunction (items 15–21), and severe depression.^22^ Higher scores on this questionnaire reflect a poorer health status.

### Self-rated health

This scale assesses self-rated health using the question: In general, how would you rate your health as poor, fair, good, very good, or excellent? Participants evaluate their health status on a 5-point Likert scale.^23^ Higher scores reflect an improved health status.

### Single-Item Self-Esteem Scale

Self-esteem was assessed using the Single-Item Self-Esteem Scale.^24^ This measurement uses a 5-point Likert scale, where higher scores reflect greater self-esteem. It serves as a valuable tool for assessing self-esteem and has been widely employed in research studies.^25^^,^^26^

### Childhood Trauma Questionnaire

The Childhood Trauma Questionnaire is a self-report tool consisting of 28 items, designed to assess 5 dimensions of childhood trauma: sexual abuse, emotional abuse, physical abuse, emotional neglect, and physical neglect. The 3 items in this questionnaire assess whether the participant aims to reduce the challenging experiences within the family. The initial version and extended format of this questionnaire were introduced in 1994,^27^^,^^28^ with its shorter version released in 2003.^29^ This questionnaire has undergone psychometric evaluation across various populations, demonstrating its reliability and suitability for assessing childhood trauma.^29^, ^30^, ^31^ Among adolescents, the Cronbach's alpha coefficients for each subscale are as follows: emotional abuse (α = 0.89), physical abuse (α = 0.86), sexual abuse (α = 0.95), emotional neglect (α = 0.89), and physical neglect (α = 0.78).^29^ The questionnaire is scored using a 5-point Likert scale, ranging from 1 (never true) to 5 (very often true).^29^ “Moderate-severe cutoff scores for each subscale are ≥ 13 for Emotional Abuse; ≥ 10 for Physical Abuse; ≥ 8 for Sexual Abuse; ≥ 15 for Emotional Neglect; and ≥ 10 for Physical Neglect ”.^32^

### Statistical analysis

Initially, the descriptive characteristics of the participants, including percentages, were presented for each group. Subsequently, the mean and standard deviation of the outcome variables were reported for the case and control groups. To examine the differences in outcome variables between these groups, a multivariate analysis of covariance was conducted. The analysis accounted for control variables such as age, education, economic status, alcohol consumption, disease history, physical activity, body mass index, and smoking habits. Multivariate covariance adjustments were performed with 95% confidence intervals. Data analysis was carried out using SPSS version 26 (IBM, USA).

## Result

**Figure 1:**
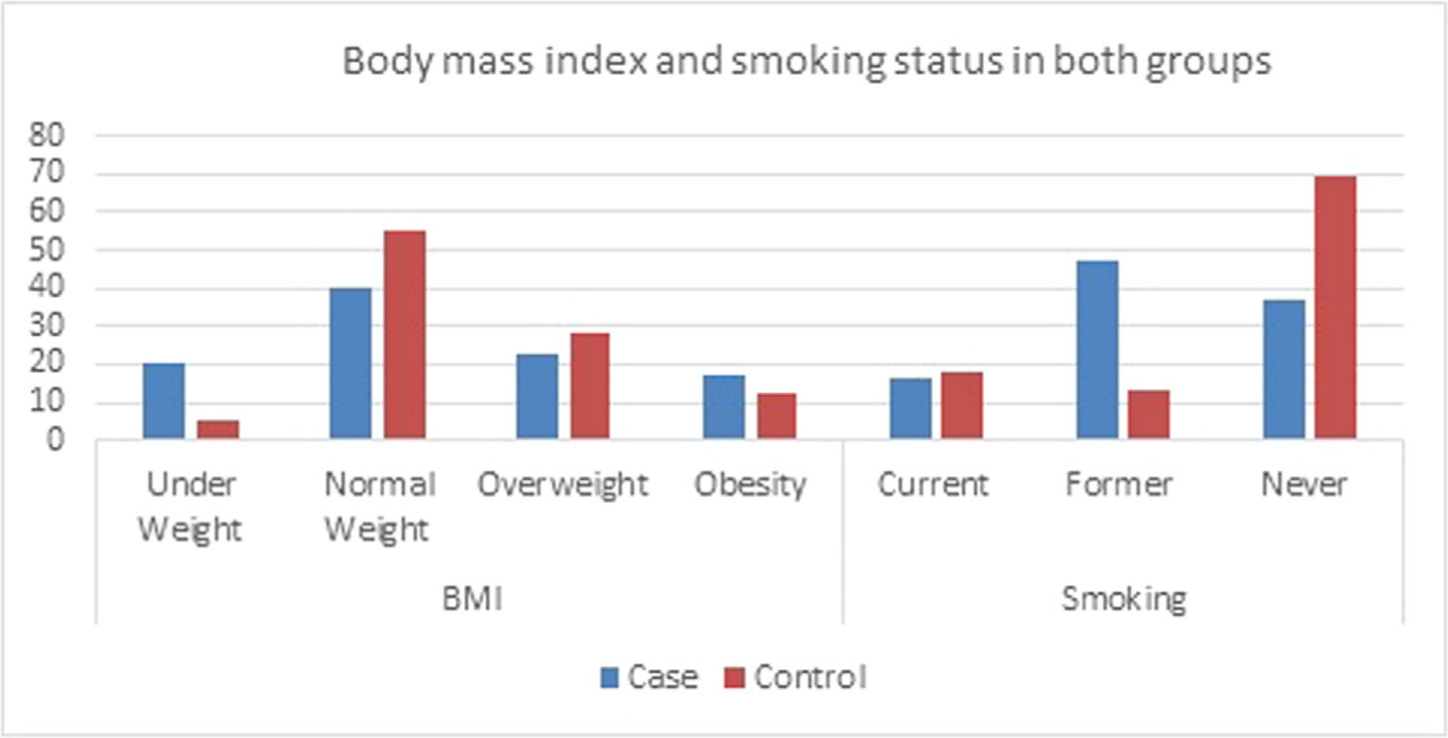
Body mass index and smoking status in both groups.

Supplementary Table 1 provides the demographic details of the participants, while Figure 1 illustrates the prevalence of overweight, obesity, and smoking. Among the total number of individuals seeking cosmetic surgery, the specific procedures requested included rhinoplasty (27 cases), facelifts (25 cases), and eyelid surgery (21 cases). The remaining 27 individuals expressed interest numerous cosmetic procedures, such as liposuction, breast augmentation, chin surgery, and skin resurfacing.

**Figure 2:**
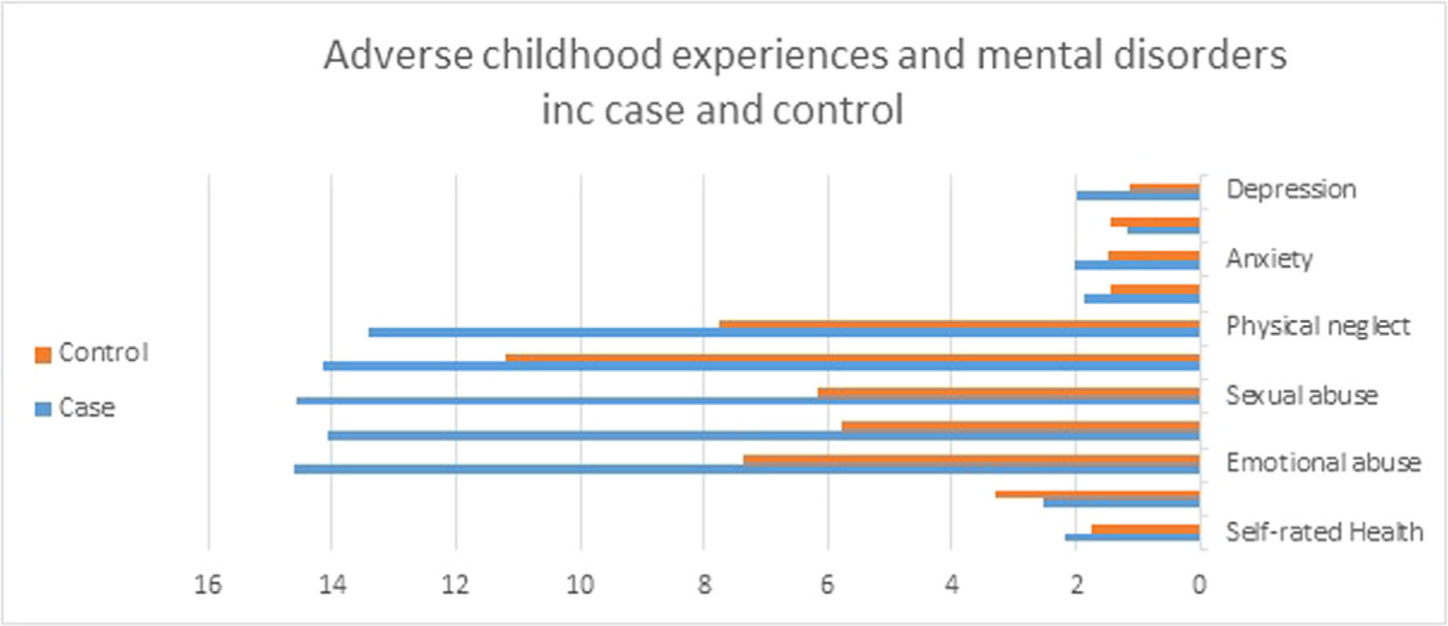
Adverse childhood experiences and mental disorders inc case and control.

Table 1 provides the mean and standard deviation data for the cosmetic surgery-seeking and control groups across variables such as ACEs, mental disorders, health assessment, and self-esteem. The results revealed that individuals seeking cosmetic surgery scored higher in ACEs and in all components of mental health issues, with the exception of social dysfunction (Figure 2).

**Table 1:** Mean and standard deviation of adverse childhood experiences and mental health issues.

Group	Case		Control		
	Mean	SD	Mean	SD	
	Self-rated Health	2.17	.68	1.75	.59
	Self-esteem	2.54	1.14	3.28	.80
	Emotional abuse	14.61	4.90	7.36	3.12
	Physical abuse	14.06	5.80	5.77	1.36
	Sexual abuse	14.59	5.53	6.17	2.36
	Emotional neglect	14.16	3.94	11.21	4.95
	Physical neglect	13.43	3.64	7.76	2.91
	Somatic complaints	1.86	.56	1.44	.64
	Anxiety	2.01	.76	1.48	.82
	Social dysfunction	1.15	.54	1.44	.51
	Depression	2.00	.78	1.14	.84

Table 2 shows the results of multivariate covariance analysis comparing the cosmetic surgery-seeking and control groups. The obtained result shows that there is a significant difference between the 2 groups in terms of ACEs and mental health issues. In the following, these differences are examined.

**Table 2:** Multivariate tests comparing 2 groups of cosmetic surgeries and control group.

	Value	F	Hypothesis df	Error df	Sig.
Pillai's trace	.575	22.112*	11.000	180.000	*.000*
Wilks' lambda	.425	22.112*	11.000	180.000	*.000*
Hotelling's trace	1.351	22.112*	11.000	180.000	*.000*
Roy's largest root	1.351	22.112*	11.000	180.000	*.000*

⁎ P value

Table 3 illustrates that, across all the variables analyzed in this study, there is a significant difference between the group seeking cosmetic surgery and control group.

**Table 3:** Univariate Tests comparing the cosmetic surgery-seeking and control groups.

Dependent Variable	Sum of Squares	df	Mean Square	F	Sig.*
Self-rated health	3.515	1	3.515	9.269	*.003*
Self-esteem	21.280	1	21.280	22.040	*.000*
Emotional abuse	2135.581	1	2135.581	125.465	*.000*
Physical abuse	2758.183	1	2758.183	155.160	*.000*
Sexual abuse	2852.153	1	2852.153	162.201	*.000*
Emotional neglect	360.459	1	360.459	17.968	*.000*
Physical neglect	1241.404	1	1241.404	118.101	*.000*
Somatic complaints	7.923	1	7.923	22.529	*.000*
Anxiety	11.919	1	11.919	19.167	*.000*
Social dysfunction	3.811	1	3.811	13.520	*.000*
Depression	31.857	1	31.857	48.049	*.000*

⁎ P value

A pairwise comparison between the cosmetic surgery-seeking and control groups revealed that those seeking cosmetic surgery scored significantly higher in ACEs. These included emotional abuse (P < 0.001), physical abuse (P < 0.001), sexual abuse (P < 0.001), emotional neglect (P < 0.001), and physical neglect (P < 0.001). The group pursuing cosmetic surgery reported better self-rated health (P = 0.003) compared to the control group. However, a comparison of self-esteem revealed that this group exhibited lower levels of self-esteem (P < 0.001) than the control group. When examining mental health and its components, the findings showed that individuals seeking cosmetic surgery experienced higher levels of somatic complaints (P < 0.001), anxiety (P < 0.001), and depression (P < 0.001) relative to the control group. Social dysfunction (P < 0.001) was significantly lower in the group seeking cosmetic surgery compared to the control group, as presented in Table 4.

**Table 4:** Comparing the cosmetic surgery-seeking and control groups.

Dependent Variable	(I) Group	(J) Group	Mean Difference (I-J)	Std. Error	Sig.**	95% Confidence Interval for Difference	
						Lower Bound	Upper Bound
Self-rated health	Case	Control	.297*	.098	*.003*	.105	.490
Self-esteem	Case	Control	−.731*	.156	*.000*	−1.038	−.424
Emotional abuse	Case	Control	7.322*	.654	*.000*	6.033	8.612
Physical abuse	Case	Control	8.321*	.668	*.000*	7.004	9.639
Sexual abuse	Case	Control	8.462*	.664	*.000*	7.151	9.772
Emotional neglect	Case	Control	3.008*	.710	*.000*	1.608	4.408
Physical neglect	Case	Control	5.583*	.514	*.000*	4.569	6.596
Somatic complaints	Case	Control	.446*	.094	*.000*	.261	.631
Anxiety	Case	Control	.547*	.125	*.000*	.301	.793
Social dysfunction	Case	Control	−.309*	.084	*.000*	−.475	−.143
Depression	Case	Control	.894*	.129	*.000*	.640	1.149

⁎ The mean difference is significant at the .05 level.

⁎⁎ P value

## Discussion

This study used a case-control design to examine ACEs and mental health issues among individuals seeking cosmetic surgery. The findings revealed that those pursuing cosmetic procedures had a higher prevalence of ACEs compared to the general population, with a significant difference observed between the 2 groups.

This highlights the importance of studies investigating mental health conditions such as depression, anxiety, low self-esteem, and BDD among individuals seeking cosmetic surgery, which are known to have a higher prevalence of these disorders in this group.^8^ Research also indicates that they experience a greater number of ACEs. Recent meta-analysis findings indicate a strong correlation between ACEs and the development of BDD.^17^ Cosmetic surgery seekers often face more adverse experiences because ACEs can negatively impact body image. This damage can result in a distorted perception of one's own body, leading the individual to continuously seek ways to compensate for this. Studies have indicated that ACEs can negatively impact social, cognitive, and emotional development, while also promoting behaviors that pose risks to health and overall well-being.^33^ ACEs are linked to physical health challenges, mental health issues, low self-esteem, and a disrupted perception of body image.^34^, ^35^, ^36^, ^37^ Conversely, the increased prevalence of alcohol consumption and smoking among individuals with ACEs,^38^^,^^39^ may suggest that these behaviors served as coping mechanisms. Mental health issues observed in individuals seeking cosmetic surgery are not merely superficial. It is essential to address the underlying factors contributing to such struggles. Experiences such as adverse events during childhood can have profound and lasting impacts on mental health, often emerging in various forms across different aspects of social and personal life.

To the best of our knowledge, this research is the first to examine mental health and explore the impact of ACEs in individuals seeking cosmetic surgery. Thus, it provides valuable insights that could enhance our understanding of how childhood traumas influence various physical and psychological aspects over the long term. However, there are certain limitations in this study that warrant attention in future research. As this was a cross-sectional study involving candidates for cosmetic surgery, assessments were conducted prior to the procedures. Longitudinal studies are needed to investigate the effects of cosmetic surgery on mental health over time. Additionally, future studies should incorporate diverse cultural contexts to better understand cultural and media influences on the pursuit of cosmetic surgery. This study relies on self-reported data, which inherently presents biases, particularly in sections addressing ACEs. Furthermore, the national social, domestic, and political contexts of different countries significantly influence how individuals perceive and respond to the prospect of cosmetic surgery. It is also important to acknowledge that beyond the outcome variables and confounding factors analyzed in this research, numerous cultural, personal, and health-related elements might impact the results. These factors warrant further investigation in future studies. A key point to consider about the findings of this study is that it was conducted as a single-center study in Iran. Consequently, caution is necessary when attempting to generalize the results. To address this limitation, future research should adopt a multicenter approach. Additionally, similar studies should be carried out in other countries to broaden the scope of understanding. Such efforts would significantly contribute to expanding the knowledge in this area.

## Clinical implications

This study revealed that individuals seeking cosmetic surgery tend to have faced more ACEs compared to the general population and are also more likely to encounter challenges related to mental health issues. ACEs appear to be a potential risk factor for pursuing cosmetic surgery later in life. Consequently, it becomes essential to approach mental health assessments in greater depth and precision during presurgery evaluations. Ignoring these aspects could lead to dissatisfaction with the outcomes of cosmetic procedures, which may have notable implications for the patient and surgeon. Focusing on the mental health issues of individuals considering cosmetic surgery can play a significant role in minimizing adverse psychological outcomes. Incorporating psychological screening and counseling as mandatory steps in cosmetic surgery clinics could enhance client satisfaction and help decrease the prevalence of unnecessary procedures.

## Informed consent

Informed consent was obtained from all subjects.

## Ethical approval statement

This project has been approved as a master's thesis.

## Conflict of interest

There is nothing to declare.

## Appendix Supplementary materials

Image, application 1
