# Supplementary material for: Adverse childhood experiences and mental health issues in patients seeking cosmetic surgery: A case-control study
Source: JPRAS Open. 2025 Mar 25;44:339–47. doi: 10.1016/j.jpra.2025.03.010 (PMC12035720; doi:10.1016/j.jpra.2025.03.010)
Supplement: Supplementary file 1 [file mmc1.docx]

| **Supplementary Table1.** Descriptive indicators of participants in the case and control groups | | | | |
| --- | --- | --- | --- | --- |
| Variable | | Total | Case | Control |
|  |  | Percent | Percent | Percent |
| BMI | Under Weight | 12.5 | 20.0 | 5.0 |
|  | Normal Weight | 47.5 | 40.0 | 55.0 |
|  | Overweight | 25.5 | 23.0 | 28.0 |
|  | Obesity | 14.5 | 17.0 | 12.0 |
| Education | Diploma and lower | 19.0 | 25.0 | 13.0 |
|  | Bachelor's | 44.0 | 48.0 | 40.0 |
|  | Master's | 37.0 | 27.0 | 47.0 |
| Economic | Very poor | 2.0 | 1.0 | 3.0 |
|  | Poor | 10.0 | 13.0 | 7.0 |
|  | Moderate | 32.0 | 31.0 | 33.0 |
|  | Good | 48.5 | 46.0 | 51.0 |
|  | Very good | 7.5 | 9.0 | 6.0 |
| Smoking | Current | 17.0 | 16.0 | 18.0 |
|  | Former | 30.0 | 47.0 | 13.0 |
|  | Never | 53.0 | 37.0 | 69.0 |
| Alcohol use | Yes | 32.5 | 51.0 | 14.0 |
|  | No | 67.5 | 49.0 | 86.0 |
| History of Disease | Yes | 35.5 | 35.0 | 36.0 |
|  | No | 64.5 | 65.0 | 64.0 |
